# The impact of sleep in high-risk infants

**DOI:** 10.1038/s41390-025-04049-2

**Published:** 2025-04-10

**Authors:** Ann-Cathrine Neukamm, Mirja Quante, Christian F. Poets, Renée A. Shellhaas

**Affiliations:** 1 Department of Neonatology, University of Tuebingen, Tuebingen, Germany; 2 Department of Neurology, Washington University School of Medicine, St. Louis, MO USA; 3 Neonatal Intensive Care Unit, Cambridge University Hospitals NHS Foundation Trust, Cambridge, UK; 4 Paediatrics and Child Health, Cork University Hospital, Cork, Ireland; 5 INFANT Research Centre, University College Cork, Cork, Ireland; 6 Department of Development and Regeneration, University Hospitals Leuven, Neonatal Intensive Care Unit, KU Leuven Leuven, Belgium; 7 Neonatal Intensive Care Unit, UZ Leuven, Leuven, Belgium; 8 Department of Neonatology, University Medical Center Utrecht, Wilhelmina Children’s Hospital, Utrecht, the Netherlands; 9 Dynamics of Youth, Utrecht University, Utrecht, the Netherlands

## Abstract

**Abstract:**

Most of an infant’s day is devoted to sleep – and normal sleep is vital to normal brain development. Sleep disruptions may impair overall health, well-being, and neurodevelopment. Disruptors of sleep and circadian health, such as noise, light, respiratory support, and clinical interventions, are highly prevalent in hospital and nursing care facilities. These factors particularly affect infants who already have an increased risk of sleep disorders and their consequences due to an underlying disease. Preterm infants and infants with disorders such as neonatal abstinence syndrome, craniofacial malformations, congenital heart disease, hypoxic-ischemic encephalopathy, Chiari-malformation/myelomeningocele, congenital musculoskeletal disease, and Down syndrome are all at high risk for impaired development of sleep-wake cycling and for sleep-disordered breathing. Since abnormal sleep is a potentially treatable risk factor for impaired neurodevelopment, there is an urgent need for effective monitoring, timely interventions, and treatment strategies to improve sleep physiology and thereby optimize overall neurodevelopment in these high-risk populations.

**Impact:**

- Healthy sleep plays a fundamental role in normal infant brain development.
- Many factors can disrupt sleep during a hospital stay. This is particularly important for infants who have an increased risk of sleep disorders due to neonatal disorders such as prematurity, congenital heart disease, or Chiari malformation.
- Sleep protective strategies are readily available and need to be systematically implemented into hospital care.

## Introduction

Sleep is essential for memory consolidation, synaptic plasticity, and the formation of neural connectivity, all of which are important for normal neurodevelopment.^1–5^ There is much evidence that children who experience inadequate or disrupted sleep show a higher risk of developmental delays, behavioral problems, and difficulties in learning and memory.^6,7^ For example, higher proportions of total night-time sleep at 18 and 26 months were related to better performance in executive tasks a few months later.^8^ In neonates at risk for seizures, inefficient sleep, expressed as a higher proportion of quiet sleep and lower electroencephalogram (EEG) delta power during this stage, was associated with worse neurological examination scores and predicted worse 18-month cognitive, language and motor scores.^9,10^

Beyond sleep-wake cycling (SWC) as a marker of infant brain function, sleep-disordered breathing (SDB) may be a remediable contributor to unfavorable neurodevelopment. Neonates who require Neonatal Intensive Care Unit (NICU) admission commonly have SDB.^11^ Likewise, newborns with craniofacial anomalies, certain genetic syndromes, neuromuscular diseases, and other congenital anomalies are also at particularly high risk.^12^ The risk for SDB is also elevated in preterm born infants.^13–15^ Here, repeated, brief, intermittent hypoxemia and resultant sleep disruption caused by SDB could exacerbate the already elevated long-term risk for abnormal cognitive development.

In addition, impaired sleep may have somatic consequences, such as reduced growth.^16^ Among Singaporean children in the first two years of life, shorter sleep duration was significantly associated with shorter body length and, in some subgroup analyses, with higher body mass index (BMI).^17^ There is strong evidence from studies in older children and adults that a short sleep duration is associated with an increased risk of obesity, and in addition, adverse sleep characteristics may affect glucose and insulin metabolism and blood pressure, and thus overall cardiovascular risk factors.^18^

This narrative review aims to give an overview of the current knowledge base of sleep disruptors and how these factors particularly affect infants during their first year of life - a demographic with an increased risk of sleep disorders and their consequences due to an underlying disease.

## Role of sleep disruptors

**Fig. 1:**
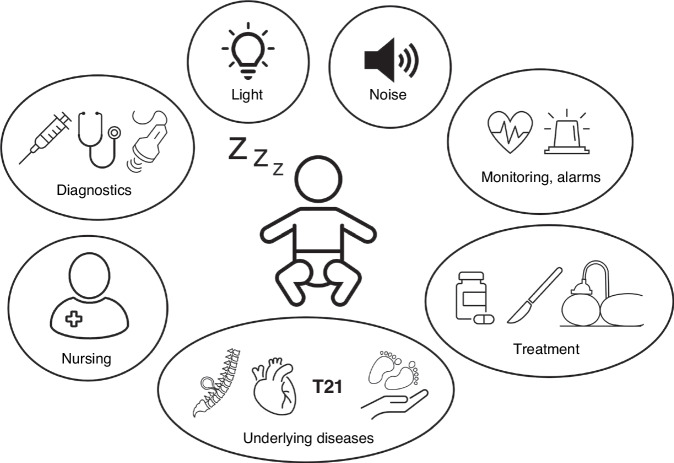
Factors influencing infants’ quality of sleep during their NICU stay. The infant at risk in its environment of possible sleep disruptors: Light, noise, monitoring and alarms (especially in NICUs/PICUs), diagnostic procedures such as blood takes, physical examinations and ultrasound, nursing activities, the underlying disease itself e.g. myelomeningocele, congenital heart disease, Down syndrome, prematurity, and treatments such as medication e.g. sedatives, surgery, mechanical ventilation.

Intensive care units are places with constant activity, alarms, and medical equipment that generates noise and light. Children undergo medically necessary invasive procedures, such as blood draws, or unpleasant interventions, such as physical examinations, ultrasound scans or X-rays and nursing activities. See Fig. 1 for a graphical summary of possible sleep disruptors (Fig. 1). In the following sections, we will provide an overview of selected sleep disruptors.

### Noise

Noise measurements in incubators in NICUs showed means of 53–62 dB with maximum levels of up to 113 dB, with no major differences between day and night.^19–21^ These sound levels are frequently above the threshold recommendations of the American Academy of Pediatrics (AAP), which recommends 45 dB as the maximum sound exposure with sound peaks at or below 65 dB^22–24^ and the World Health Organization (WHO), which recommends an even lower maximum of 30 dB for hospital ward rooms during the night.^25^ Ventilatory support significantly increases the background noise; measurements showed a variable noise level depending on the ventilator, ventilation mode, and precise microphone positioning used, but always between 45 dB and up to 82 dB.^26^

In preterm infants, noise peaks above 65 dB led to blinking and a startle reflex, facial and body movements in the majority of newborns, as well as changed sleep and wake states in 60%.^21^ In contrast, others reported no significant correlation between noise levels and wakefulness in preterms.^19^ One study group performed a period of “quiet time” four times a day for 60 minutes each in a NICU. They found that neonates with a corrected age of 35 weeks’ gestation had longer total sleep time during the 60-minutes “quiet time”, followed by a longer bout of wakefulness.^27^

There also seems to be a correlation between sound exposure in preterm infants and neurodevelopmental outcomes. A study of 34 preterm infants indicated an association between sound reduction by silicone earplugs and a better mental development index at 18–22 months.^28^ Here, sleep seems to be the mediator linking noise exposure to poor recovery and adverse neurodevelopmental outcomes. Conversely, there is concern that limiting sound exposure in NICUs could limit necessary exposure to spoken language with long-term consequences for language development. A study comparing auditory exposure in NICU private rooms and in the open ward, found significantly more silent time in the private rooms (1.9 h during a 16h-period)^29^ and another study described lower language scores in 2-year-old children who were previously hospitalized in NICU private rooms compared to children with previous hospitalization in an open ward.^30^ Furthermore, exposure to parental talk increased infant vocalization in preterm infants.^31^ Moreover, live music therapy, especially lullabies, has a positive impact on physiological functions in preterm infants with lower heart and breathing rates, better caloric intake, and improved sleep patterns.^32–34^ Thus, not only noise reduction but also the promotion of positive sounds, such as spoken language and music, seems to be important.

### Light

Light exposure in intensive care units is highly variable. On the one hand, light is necessary to carry out interventions safely and to assess children clinically; on the other hand, children should not be dazzled or disturbed. The AAP and a consensus committee, therefore recommend variably adjustable light sources that provide indirect lighting and ambient light in a range of 10 to 600 lux.^23,24^ A study in extremely low birth weight infants measured a mean light level of 70.5 lux throughout their hospital stay (excluding phototherapy episodes), and light levels almost always met the AAP recommendations.^35^ Others reported light levels more than twice the recommended levels at multiple times of the day in the environment of a neonate with chronic heart disease,^36^ while another study found light levels generally below the recommended values in infants with chronic heart disease.^37^

Interestingly, high light levels achieved during phototherapy can also alter sleep behavior after the end of the exposure. A large Japanese study found shorter sleep time over 24 hours at one month of age in children who had received phototherapy over 24 hours.^38^ It is increasingly recommended to adapt the light to day and night time in intensive care units to support the existing or developing circadian rhythm.^39,40^ Even in preterm and newborn infants, positive effects of light cycling have been shown with shorter length of stay, better weight gain and less crying at 11 weeks’ corrected age.^41,42^

Overall, optimizing light exposure in the intensive care unit environment seems beneficial for the development of infants and sleep health. However, a recent review emphasized the low level of evidence supporting chronobiology in NICUs.^43^

### Interventions by nurses and physicians

In many intensive care units, an attempt is made to take the infant’s sleep-wake cycle into account and adapt interventions accordingly. However, there is a risk that active sleep may be mistaken for wakefulness due to the presence of frequent body and facial movements, and the infant may thus be disturbed in its most vulnerable sleep state.^44–46^ Active sleep plays a particularly important role in brain development, especially sensorimotor plasticity, which is underscored by the fact that preterm infants, during a period of extremely rapid development, spend more time in active sleep than older children.^47,48^ Additionally, SDB is typically exacerbated during this sleep stage, and respiratory events are most commonly induced duringthe handling of infants in active sleep.^44^ A survey of Dutch healthcare providers who were directly involved in neonatal care revealed limited knowledge of sleep physiology, with only 14% correctly answering a question about the difference between wakefulness and active sleep.^45^ Similarly, in a study of 30 Iranian nurses working in a NICU, only 20% were able to describe the characteristics of active sleep.^49^

A polysomnography (PSG) study in term and near-term infants at risk of cerebral dysfunction during their NICU stay revealed frequent handling with a total duration of hands-on care of 65.3 ± 33.0 minutes within 4 hours.^44^ These contacts, including direct contact with the neonate and with objects within the incubator and, in 34% of cases, with the PSG-related technology, occurred in equal proportions during all sleep and wake stages with a mean maximum time interval between contacts of 50.9 ± 26.2 minutes. Only half of the neonates were able to complete a 60-minute sleep-wake cycle without interruption.^44^ Another study in 12 preterm infants with a corrected age of 35 weeks’ gestation observed a mean of 143 handlings (of which 55 were due to adjustments of polysomnography) for each infant with a total time of 3.9 h in 24 hours with more handlings during the day, but there was no statistically significant association between number and duration of handling episodes and wakefulness.^19^ Two small postoperative studies in neonates with chronic heart disease also found frequent arousals and only a short duration of sleep episodes with an average of 12.3 minutes^37^ and sleep durations of less than 30 minutes for 90% of the time.^36^ In an Irish study of moderate to late preterm infants 23% of overnight sleep cycles were interrupted, almost exclusively for the purposes of feeding.^50^

Additionally, experiencing procedural pain is common in NICUs or Pediatric Intensive Care Units (PICUs). One study reported a median of 35 skin breaking procedures in the first five days in a group of preterm infants, which showed a significant correlation with maturation on EEG measured with polysomnography at discharge.^51^ Another study showed that higher levels of stress, as measured by salivary cortisol, prolonged periods of wakefulness.^52^ Interestingly, this group also found that very and moderately preterm infants were highly resilient to nociceptive stimuli during active sleep and did not awaken, whereas older neonates (34 – 40 PMA) were particularly vulnerable during active sleep and often awakened to the same stimuli.^52^

In summary, many factors in the hospital environment can disrupt sleep. It is important to optimize the local clinical environment to promote sleep and thus development during a vulnerable period of life. In the following sections, we will take a closer look at some specific patient groups.

## Preterm infants

Preterm infants spend up to 90% of their time asleep.^53^ There is some evidence that preterm infants experience structurally different sleep, e.g., longer sleep cycles, more wakefulness, and higher proportions of quiet sleep at similar post-menstrual ages, demonstrating that being born preterm impacts sleep regulation.^54–56^ This is consistent with the finding that babies born preterm are at significant risk of impaired brain development, which may manifest as altered sleep regulation.^57^ In addition, significant disruptions in sleep patterns in preterm infants are common and may exacerbate atypical brain development and increase the risk of developing severe cognitive, behavioral, and/or socialization deficits.^9,58,59^

As outlined earlier, the NICU could be considered a hostile sleep environment for preterm infants. Apart from the external disturbers, inherent pathologies and comorbidities themselves have the potential to hinder normal sleep in this population. In this regard, rapid eye movement sleep (REM) -type sleep plays a particular role. For example, a recent study showed an association between active sleep percentage during 29–32 weeks’ postmenstrual age and white matter volume at term age.^60^ Additionally, infants with more REM-type (active) sleep proportions also had better neurodevelopmental outcomes as assessed via Bayley II.^58^

Even after the NICU stay, many former preterm infants still exhibit abnormal sleep features with lower sleep quality and more parental worries about their baby’s sleep.^61^ Recently published recommendations highlight eight evidence-based practices to protect and promote sleep; as well as noise and light control discussed earlier, emphasis should be placed on sleep team composition, risk factor assessment, sleep assessment tools, positional management, sensory stimulation, and hospital-home transition sleep management.^52,62^

## Neonatal abstinence syndrome

Neonatal abstinence syndrome (NAS), or more specifically neonatal opioid withdrawal syndrome in the case of maternal opioid use, refers to a group of symptoms that occur in newborns who have been exposed to addictive substances in utero. Symptoms can include irritability, tremors, feeding difficulties, vomiting, diarrhea, and specifically sleep disturbances.^63^ A large proportion of children (39–100%) with NAS showed no SWC during amplitude-integrated electroencephalography (aEEG) monitoring in the first 1–4 postnatal days before medical treatment^64–66^ compared to only 8% of healthy controls.^65^ Abnormal aEEG patterns and lack of SWC were associated with higher NAS scores and a longer hospital stay.^64,66^

Compared to healthy infants, those with NAS spend more time in active sleep^67,68^ and less time in quiet sleep.^67–69^ Additionally, sleep of infants with NAS is more disorganized with a higher percentage of indeterminate sleep^68,69^ and a higher frequency of change in sleep patterns.^67^ Overall, infants with symptoms of NAS spent more time awake than controls and, therefore, had poorer sleep efficiency, but when treated with oral morphine, sleep efficiency improved.^68,69^

There have been several attempts to improve sleep in children with NAS. However, sleeping in a rocking bed increased withdrawal symptoms and sleep disruption on day seven compared to sleeping in a normal bed.^70^ Another study compared sleeping in a prone position with sleeping in the supine position. Neonates in the prone position showed significantly lower mean and peak withdrawal scores and lower daily caloric intake.^71^ However, sleeping in the prone position has to be considered very critically because of its increased risk of sudden infant death syndrome (SIDS).^72^ Promoting sleep in infants with NAS is particularly important, as children with NAS are already at increased risk of neurocognitive impairment.^73,74^

## Craniofacial malformations (e.g. Robin Sequence)

Because of their specific anatomy, upper airway obstruction (UAO) is particularly common in neonates, but not always easy to detect at this age. In some infants, UAO only develops some weeks after birth, thus, a normal sleep study result shortly after birth does not preclude the development of potentially severe obstructive sleep apnea (OSA) during the first postnatal months.^75^ Also, the common belief that sleep-related UAO will lead to frequent oxygen desaturations is not always true.^76^ In fact, some children have severe OSA on PSG but no clinical symptoms or desaturations, so that pulse oximetry is not sensitive enough to reliably detect OSA in this population.^77^

Even if the pathophysiology of UAO is evident, as in infants with RS, the expression of mandibular retrognathia or glossoptosis as the main symptoms correlate only poorly with the severity of UAO/OSA,^78^ which justifies routine screening for OSA in all infants with these conditions. If no PSG is available, cardiorespiratory polygraphy (PG), but not oximetry, may be used as a valid alternative.^79^ Using a PG will result in similar values for the mixed obstructive apnea hypopnea index but will detect hypopneas less often than with PSG.^80^ In a study on infants with RS, the mixed-obstructive apnea-hypopnea index was twice as high as the mixed-obstructive apnea index.^81^ Given that recording a full PSG is more burdensome on these infants, which may already be severely compromised by their respiratory problems, it may be acceptable only to record a PG for infants with RS, even if full PSG is available, but to reduce treatment thresholds. For example, in the authors’ setting, a threshold of 3 instead of 5 events/h is used.^75^ Assessing the efficacy of treatment also requires a sleep study. Thus, this diagnostic modality should be available in every center where infants with craniofacial malformations are treated.^82^

Of particular concern in this context is the frequently applied practice of using the prone sleep position as a first-line treatment for infants with craniofacial malformations. This is because of the large body of evidence identifying this position as one of the most important preventable risk factors for SIDS,^72^ and although a death in an infant with RS would not be classified as SIDS, there is no reason to suggest that a similar pathophysiology as seen in healthy infants would not also apply to infants with RS. In addition, there is accumulating evidence that prone positioning is not effective in most infants with RS.^83^ Thus, given the combination of limited effectiveness plus an increased risk of sudden death precludes, in our opinion, a recommendation for prone sleep positioning in this patient group.^84^ This is especially true given that effective non-surgical treatment options exist for these infants, although not yet in every country.^82,85,86^

Little is yet known about the clinical consequences of recurrent obstructive apneas as seen in infants with craniofacial malformations. While there is little doubt that frequent respiratory events may result in severe failure to thrive (as seen in infants with RS),^87^ obstructive apneas were also reported as one of the few sleep study results differentiating infants who later succumbed to SIDS from healthy controls.^88^ An explanation for this finding may lie in the fact that recurrent obstructive apneas, e.g., in RS infants, are associated with an increased arousal threshold, with an impaired arousal also being implied in the pathophysiology of SIDS.^89,90^ In addition, there is evidence that these patients may have a normal neurocognitive development if their sleep-related upper airway obstruction is identified and treated early,^86,91,92^ further supporting the relevance of routinely performing sleep studies in this high-risk group.

## Congenital heart disease

Sleep physiology is often disturbed in children with congenital heart disease (CHD). In EEG observations, children with CHD showed a lower proportion of active sleep and a shorter sleep cycle duration compared to healthy term neonates.^93^ Later, at 6–12 months of age, cyanotic CHD infants had increased wake time with decreased sleep efficiency.^94^ One study reported a calculated functional brain age (FBA) based on the distribution of quiet sleep and non-quiet sleep as an expression of brain maturation.^93^ They found a lower FBA in children with CHD, and even more in children with dextro-transposition of the great arteries than in healthy neonates. Postoperatively, in this study, sleep organization and FBA improved and no longer differed from healthy controls.^93^ Several studies report disturbed SWC in up to half of children with CHD before corrective surgery,^95–98^ whereas other studies found normal SWC preoperatively in a high proportion of children with CHD (87–97%)^99–101^ Table 1 provides a summary of available literature on sleep wake cycling in infants with congenital heart disease (Table 1). Most of these studies performed aEEG monitoring in the first days of life, with the exception of one study with measurements just before surgery at a mean age of 1.4 months;^95^ the different findings could not be explained by differences in age.

**Table 1:** Sleep wake cycling in infants with congenital heart diseases.

Reference	Sample size	Age at birth	Age at surgery/EEG	Type of CHD	Method of monitoring	SWC preoperative	SWC postoperative	Neurocognitive outcome
Claessens et al.^97^	76	>36 PMA, no genetic disorders or multiple congenital anomalies	Neonatal period (surgery)	CHD with neonatal surgery with CPB	aEEG, 6 h before surgery, ≥ 36 h after surgery, additionally in 29 neonates shortly after birth	48% normal SWC preoperative 34% normal SWC within 24 h after birth	29% normal SWC within 48 h	no association between lack of recovery of SWC and new brain injury in MRI
Gui et al.^95^	103	≥PMA 37, no genetic abnormalities	mean age of 1.4 months (surgery)	CHD with surgery within 3 months	aEEG, ≥ 24 h 1 -2 days before and 3 – 7 days after surgery	60% normal SWC	16% better SWC 68% no change 17% worse SWC	–
Gunn et al.^102^	150	≥PMA 36, no genetic abnormalities associated with impaired neurodevelopment	mean age of 7 days (surgery)	CHD with surgery before 2 months	aEEG, 1 h preoperative, intra- and 72 h postoperative	–	79% SWC within 72 h (median 21 h)	Association between delayed SWC recovery and increased mortality and worse BSID-III scores at 2 years
Hermans et al.^93^	15 CHD 24 controls	≥36 PMA, no genetic malformation syndrome	1-6 days of age (preoperative recordings), 7-16 days (postoperative recordings, after intensive care period)	8 d-TGA vs 7 other CHD vs 24 healthy neonates	cEEG, calculation of Functional Brain Age (FBA) based on the distribution of quiet sleep and non-quiet sleep	FBA 37.2 weeks (d-TGA), FBA 39.3 weeks (non-TGA CHD), FBA 38.2 weeks (controls)	No differences in FBA between CHD and controls	Association between FBA delay and lower BSID-III motor scores at 2 years
Latal et al.^100^	60	mean PMA 39.45 (30.6-41.9), no genetic comorbidity or suspected syndromic disorder	mean age of 10 days (surgery)	CHD with CPB surgery within 3 months	aEEG, 12 h pre- and 48 h postoperative	92% normal SWC	71% return to normal SWC	lack of return to SWC predicted poorer IQ at 4 years but not motor outcome
Mebius et al.^101^	72	≥36 PMA	first 72 h after birth (aEEG)	prenatally diagnosed CHD	aEEG, ≥ 24 h	95% normal SWC within 36 h 97% normal SWC within 72 h	–	–
Mulkey et al.^98^	24	≥36 PMA, no major genetic syndrome	aEEG after birth (mean 0.71 days)	CHD with surgery in first 30 days	aEEG, 24 h	33% SWC at least half of the recording 50% SWC at some point	–	Preoperative MRI: no association between SWC and brain injury. Of 8 infants with brain atrophy only 1 with SWC
Padiyar et al.^99^	77	≥36 PMA, no genetic disorders or multiple congenital anomalies	median age of 6 days (surgery)	CHD with CPB surgery within first 6 months of life	cEEG preoperative, 72 h postoperative	87% normal SWC	28% normal SWC immediate post-operative, 33% normal SWC 24 h postoperative	–
Ter Horst et al.^109^	62	≥36 PMA	aEEG at mean 7.5 h after starting prostaglandin infusion	24 cyanotic CHD, 38 acyanotic CHD	aEEG, 72 h	58% normal SWC, no difference between acyanotic and cyanotic, SWC more frequent in CoA than in HLHS (92% vs 48%)	–	–

*aEEG* Amplitude integrated EEG, *AS* aortic valve stenosis, *BSID-III* Bayley Scale of Infant Development-III, *cEEG* continuous EEG, *CHD* congenital heart disease, *CoA* aortic coarctation, *CPB* cardiopulmonary bypass, *d-TGA* dextro-Transposition of the great arteries, *EEG* Electroencephalography, *FBA* Functional Brain Age, *h* hours, *HLHS* hypoplastic left heart syndrome, *IQ* intelligence quotient, *PMA* postmenstrual age, *SWC* sleep wake cycling.

Postoperatively, many infants with CHD do not return to normal SWC in the first days (normal SWC in 33% within 24 h,^99^ 29% within 48 h,^97^ and 32% within 48 h ^100^). Delayed recovery of SWC after surgery was associated with poorer neurocognitive outcome at 2 years^102^ and lower intelligence quotient at 4 years.^100^ FBA delay was associated with poorer motor scores at 2 years, but not cognitive and language scores.^93^

There is some evidence that SDB is also more likely to occur in infants with CHD.^94,103,104^ In infants with CHD, comorbid SDB was associated with poorer neurocognitive test scores, including 10–12 points lower IQ.^105^

## Hypoxic-ischemic encephalopathy

SWC is not yet developed in many children with neonatal hypoxic-ischemic encephalopathy (HIE) in the first days after birth,^106–111^ and the severity of HIE influenced the time to develop SWC.^112^ Furthermore, sleep organization was altered in infants with HIE. Asphyxiated infants with mild to moderate encephalopathy showed an increased percentage of quiet sleep (46.5% vs 38.7%) and indeterminate sleep and a decreased percentage of active sleep (18.9% vs 44.7%) compared to healthy neonates.^113^

Therapeutic hypothermia is the standard treatment for children with moderate to severe HIE,^114,115^ but hypothermia itself may affect sleep organization. A study of neonates with moderate to severe HIE randomized to either hypothermia or normothermia treatment in clinical trials showed that neonates in hypothermia took longer to normalize aEEG patterns and to develop SWC compared to infants in normothermia.^108^

The recovery of SWC can be a predictor of neurologic outcome. Asphyxiated infants with pathologic MRI findings needed longer to develop SWC than infants with HIE and normal MRI imaging.^107,111^ In addition, the time to SWC onset predicted the outcome as measured by Bayley Scales at 18–36 months^106,108,116^ or by Griffith’s developmental quotient at 12–66 months.^110^ Failure to develop SWC in the first days of life (48–120 hours) strongly predicted death or disability.^108,110,111,117–119^ Furthermore, seizures were associated with a lack of SWC^120^ or a longer time interval to onset of SWC^110^ and a prolonged time to recovery of SWC was a prognostic factor for the later development of epilepsy.^121^ These findings underline that the development of SWC reflects healthy brain function.

Infants with moderate HIE showed more SDB as well as sleep initiation and maintenance issues, whereas infants with mild HIE had more circadian rhythm disturbances.^122^ There are no data on how early family education in HIE may influence later sleep. Identifying and treating sleep problems is particularly important because the occurrence of sleep problems is significantly correlated with lower quality of life and may persist into adulthood.^123^

## Chiari-malformation & myelomeningocele

Myelomeningocele (MMC) is characterized by exposure of the spinal cord through a defect in the spine and is typically accompanied by hindbrain herniation (Chiari II malformation). Infants with myelomeningocele are at high risk of sleep disorders. Abnormal sleep physiology is likely multifactorial in this population, related to MMC level, Chiari-II malformation and resultant brainstem dysfunction, musculoskeletal factors, and pulmonary abnormalities. While fetal surgery reduces the severity of hindbrain herniation, decreases the need for ventriculo-peritoneal shunts, and increases the chances of independent ambulation,^124^ prenatal MMC closure does not improve cognitive outcomes.^125^

A large proportion of school-aged children with MMC have sleep apnea.^126,127^ Sleep apnea and hypoventilation may be highly consequential, though potentially treatable, for this patient population. Importantly, sleep apnea is associated with sudden death in young adults with MMC (relative risk 5.4, 95% CI 2.5–11.8, *p* = 0.005 for sudden death in patients with vs. without sleep apnea).^128^ Critically, SDB may be ubiquitous among neonates with MMC. In a matched cohort study of newborns evaluated with gold-standard bedside PSG, the AHI was significantly higher for 19 neonates with MMC (34 ± 22) than for 19 age-matched NICU controls who had no congenital malformations or specific risk factors for SDB (19 ± 11; *p* = 0.021).^129^ The AHI was identical among neonates with fetal versus postnatal myelomeningocele repair.

Data from the Children’s Hospital of Philadelphia (CHOP) indicate that “clinically significant apnea” was identified in 58 of 100 neonates with fetal MMC repair (vs. 36% of 78 in the Management of Myelomeningocele Study [MOMS trial]).^130^ Two additional studies have reported that more than three-quarters of infants <22 months of age had abnormal pneumograms.^131,132^ Most apneas were central rather than obstructive, perhaps because of compression and dysplasia of the brainstem related to the Chiari II malformation. Based on clinical observations and the above data, the Spina Bifida Association now recommends that “*all patients with neural tube defects [the most severe of which is myelomeningocele], whether they are symptomatic or asymptomatic, undergo polysomnography that evaluates for central apneas, hypoventilation, as well as obstructive sleep apneas“*.^133^ An ongoing study (NCT04251806) is evaluating whether SDB in neonates with MMC is associated with cognitive and language development, and persistent sleep disturbances at age 2-years.

## Congenital musculoskeletal disorders

SDB, particularly with nocturnal hypercapnic hypoventilation, is highly prevalent (42 – 71%) in children with neuromuscular disorders, depending on disease severity and progression.^134–138^ Skeletal deformities, such as scoliosis and joint contractures, often accompany neuromuscular disorders or syndromic diseases and may, in turn contribute to SDB.^139,140^ Furthermore, an association between nocturnal hypercapnic hypoventilation and scoliosis was found in children with neuromuscular disorders,^141^ and 52% of children with rare skeletal dysplasia had OSA.^142^ Additionally, sleep quality and quantity may be affected in children with neuromuscular diseases. Children with congenital muscular dystrophies have more frequent nighttime awakenings, lower sleep efficiency, shorter total sleep time, and decreased REM duration compared to healthy controls.^135^ There is a paucity of studies investigating sleep in this patient group during early infancy. However, based on existing studies in older children, it can be assumed that sleep is also significantly impaired in young infants if the disease manifests early.

## Down syndrome

One often ignored comorbidity in Down Syndrome (DS) is OSA. OSA occurs at a significantly higher rate in children with DS, and maybe already be apparent in infancy. The prevalence of OSA in infants with DS ranges from 20 to 30%.^143,144^ The increased OSA risk in this population can be explained by various predisposing factors, including their unique craniofacial anatomy with a small midface and a protruding tongue, hypotonia, obesity, adenotonsillar hypertrophy, thyroid dysfunction, gastrointestinal problems, and frequent respiratory infections.^143,145,146^ Untreated OSA in DS has been found to have a negative impact on sleep quality as well as daytime behavior, neuro-development and cardiometabolic health.^147^ Early diagnosis is needed, especially since children with DS have intellectual disabilities and higher cardiovascular risk, and OSA symptoms may often be overlooked.^148^ Therefore, guidelines recommend screening sleep studies during early infancy.^149^ Treatment options in infants with DS include CPAP or high-flow oxygen therapy, myofunctional training and oral appliances.^150–152^ In addition to DS, children with other chromosomal abnormalities, such as Prader-Willi syndrome, are also at high risk for sleep disorders and should be prioritized for evaluation and treatment.^153^

## Conclusion

Certain groups of patients are at particularly high risk of sleep disorders due to the anatomical and physiological characteristics of their underlying disease and the treatment required, which often includes prolonged hospitalization. Measures should be taken to promote sleep, especially in vulnerable infants. These may include, for example, controlling ambient noise and light, assessing risk factors, and training medical and nursing staff in recognizing the infant’s sleep stages and adjusting interventions accordingly. Parents should also be educated about healthy sleep so that they can best support their infant’s sleep and recognize sleep disorders. Additionally, screening protocols for sleep disorders should be applied in at-risk populations to allow for early treatment. More research is needed for optimal evidence to best support sleep health in at-risk infants.

## Data Availability

Data sharing is not applicable to this article as no datasets were generated or analyzed during the current study.
